# COVID-19 outbreaks among crew on commercial ships at the Port of Rotterdam, the Netherlands, 2020 to 2021

**DOI:** 10.2807/1560-7917.ES.2023.28.16.2200525

**Published:** 2023-04-20

**Authors:** Edward Gebuis, Bruno Vieyra, Rob Slegtenhorst, Saskia Wiegmans, Bas van Dijk, Thijs Veenstra, Saskia Tejland, Ewout Fanoy, Annemieke de Raad, Marion Koopmans, René de Vries, Saskia van Leeuwen-Voerman, Jane Whelan

**Affiliations:** 1 Public Health Service Rotterdam-Rijnmond, Rotterdam, the Netherlands; 2 Port of Rotterdam Authority, Port of Rotterdam, the Netherlands; 3 National Institute for Public Health and the Environment (RIVM), Bilthoven, the Netherlands; 4 Department of Viroscience, Erasmus Medical Centre, Rotterdam, Netherlands/ Pandemic and Disaster Preparedness Center, Rotterdam/Delft, the Netherlands

**Keywords:** COVID-19 Testing / statistics and numerical data, Disease Outbreaks, disease prevention and control, Ships, Naval Medicine/statistics and numerical data, Travel, Port health

## Abstract

**Background:**

During the COVID-19 pandemic, international shipping activity was disrupted as movement of people and goods was restricted. The Port of Rotterdam, the largest port in Europe, remained operational throughout.

**Aim:**

We describe the burden of COVID-19 among crew on sea-going vessels at the port and recommend improvements in future infectious disease event notification and response at commercial ports.

**Methods:**

Suspected COVID-19 cases on sea-going vessels were notified to port authorities and public health (PH) authorities pre-arrival via the Maritime Declaration of Health. We linked data from port and PH information systems between 1 January 2020 and 31 July 2021, derived a notification rate (NR) of COVID-19 events per arrival, and an attack rate (AR) per vessel (confirmed cases). We compared AR by vessel type (workship/tanker/cargo/passenger), during wildtype-, alpha- and delta-dominant calendar periods.

**Results:**

Eighty-four COVID-19 events were notified on ships, involving 622 cases. The NR among 45,030 new arrivals was 173 per 100,000 impacting 1% of vessels. Events per week peaked in April 2021 and again in July 2021, when the AR was also highest. Half of all cases were notified on workships, events occurring earlier and more frequently than on other vessels.

**Conclusion:**

Notification of COVID-19 events on ships occurred infrequently, although case under-ascertainment was likely. Pre-agreed protocols for data-sharing between stakeholders locally and across Europe would facilitate more efficient pandemic response. Public health access to specimens for sequencing and environmental sampling would give greater insight into viral spread on ships.

**Key public health message**

**What did you want to address in this study?**

Since March 2020, outbreaks of COVID-19 on board cruise liners and other sea-going commercial ships have been published, but the impact of COVID-19 on seafarers and consequent impact on major port activity globally has not been described. Here, we describe the number and size of outbreaks of COVID-19 among crew on board commercial vessels arriving at the Port of Rotterdam, the largest port in Europe.

**What have we learnt from this study?**

COVID-19 outbreaks were notified infrequently, 84 in total during the study on 1% of all vessels that arrived at port. More outbreaks occurred when Alpha- and Delta-variants were in circulation and onboard work ships, and fewer occurred on passenger vessels. Strict port entry restrictions at the start of the pandemic may have limited the scale of COVID-19 at sea, but undoubtedly, some COVID cases went unreported.

**What are the implications of your findings for public health?**

This study confirms the feasibility of using of port administrative systems for public health research. We propose specific initiatives: agree data sharing protocols in advance; make data linkage across databases easier; invest in early warning systems; improve communication systems within and between ports; and expand the EU Healthy Gateways project.

## Introduction

Since the severe acute respiratory syndrome coronavirus 2 (SARS-CoV-2) virus emerged in 2020, the subsequent COVID-19 pandemic has claimed over 6.8 million lives and more than 760 million cases have been reported worldwide [1]. To control and mitigate viral spread, countries were forced to adopt unprecedented public health measures including restriction of movement that resulted in the disruption of trade flows, supply chains and international shipping activity [2]. In 2020, world merchandise trade declined by 5.3% [3].

Pre-pandemic, around 11 billion tonnes of goods, annually valued at over 14 trillion US dollars in 2019, were transported globally by sea. This equates to ca 1.5 tonnes per capita worldwide [4]. In the European Union, the world’s largest trader of manufactured goods and services, 80% of goods by volume and 50% of goods by value are transported by sea, including essential commodities such as oil, gas, and grains, manufactured goods, cars and livestock. Much of this trade passes through the Port of Rotterdam, which is the largest seaport in Europe and the tenth largest port worldwide [5]. Throughout the pandemic, the port remained fully operational, receiving up to 95 seagoing vessels each day. In 2020, total throughput at the Port of Rotterdam was 436.8 million tonnes [6], a 6.9% reduction compared with 2019 [7]. The economic impact of COVID-19 is well documented, but the specific impact of COVID-19 outbreaks among the crews on board vessels passing through the port has not been reported. Outbreak reports on board individual cruise ships [8] and industrial vessels [9,10], passenger ships [11] and military vessels [12], highlight the challenges specific to COVID-19 management on board ships where infection prevention through isolation, quarantine, ventilation and personal protection may be difficult as respiratory (and enteric) viruses can spread rapidly [13,14].

In this article, we describe the frequency and magnitude of COVID-19 events on board ships at the Port of Rotterdam from January 2020 to July 2021, with an in-depth review of the challenges arising in outbreak control on board ship, illustrated in two case studies. We also make recommendations to improve future infectious disease event notification and response at commercial ports in the Netherlands and beyond.

## Methods

This was a retrospective, observational study using data routinely collected by the Public Health Service (PHS) and Port of Rotterdam Authority (PRA) on COVID-19 on sea going vessels at the port. The study period was between 1 January 2020 and 31 July 2021, during which, calendar periods where specific variants were known to be dominant and in community circulation were defined (date selection is detailed in Supplement S1): (i) the ‘wildtype period’ from 1 January to 1 November 2020; (ii) the SARS-CoV-2 Alpha variant (Phylogenetic Assignment of Named Global Outbreak (Pango) lineage designation (B.1.1.7*)* ‘Alpha-dominant’ period from 1 November 2020 to 1 June 2021; and (iii) the SARS-CoV-2 Delta variant (Phylogenetic Assignment of Named Global Outbreak (Pango) lineage designation (B.1.617.2) ‘Delta-dominant’ period from 1 June to 31 July 2021 (the end of data collection).

### The Maritime Declaration of Health: reporting cases of COVID-19 on board ship

From 13 March 2020 to the present, all sea-going vessels arriving at or passing through the Port of Rotterdam are required to submit a Maritime Declaration of Health (MDoH) 6–24 hours before their arrival (Supplement S2 and S3), and to directly contact the PHS or resubmit the MDoH if the health situation on board changes while in port. To comply with the 2005 International Health Regulations and the Dutch Public Health Act of 2008 [15], any illness of infectious origin suspected on board must be reported based on symptoms or an epidemiological link with a (confirmed) case.

### COVID-19 case definition and testing algorithm

COVID-19 is an infectious disease caused by the SARS-CoV-2 virus (incubation period of 3–6 days, range 2–14 days [16]). A suspected COVID-19 case was any crew member presenting with clinical criteria consistent with COVID-19 (cold-like symptoms, cough, shortness of breath, fever, acute loss of taste / smell without nasal congestion [17]) or with an epidemiological link with a (confirmed) case. This information was shared digitally by the PRA with the PHS who follow up with all vessels where COVID-19 was suspected on board ensuring that throughout the study period, all suspected cases on board ship that were notified were systematically tested by polymerase chain reaction (PCR). A case was confirmed if PCR, administered by a trained healthcare professional, was positive. All testing on board was the responsibility of the shipping company or agency, mostly at commercial laboratories organised by the shipping agents. According to national guidelines [18], contacts of a confirmed case were defined as ‘household’ (if sharing the same cabin), ‘close’ (having contact for > 15 min in 24 hours, at a distance less than 1.5 m apart) and ‘not close’ contacts (< 15 min at a distance > 1.5 m). Once a case was confirmed, public health (PH) authorities advised serial testing of contacts at 5-day intervals (PCR only). We defined a ‘COVID-19 event’ as at least one confirmed case diagnosed on board a vessel at the port. A ‘repeat event’ was recorded if 28 days had elapsed between events on board the same vessel.

### Inclusion and exclusion criteria

Only laboratory confirmed cases were included. Where sea-going vessels were already in port at the time of the first suspected case (e.g. docked for maintenance), and the ship / shipping agent made contact directly with the PHS for advice rather than file a second MDoH, these events were included. When a COVID-19 event was suspected on board, it was recorded as such in the port information system, but if these events remained unconfirmed, or were related to events that had already ended and the vessel docked successfully at port with a negative MDoH, the suspected event was excluded. Crew members testing positive post-recovery and suspected cases that subsequently tested negative or were unconfirmed were not routinely followed up and were not included. Inland waterway vessels are subject to different reporting regulations and are beyond the scope of this study.

### COVID-19 case management and outbreak control

**Figure 1:**
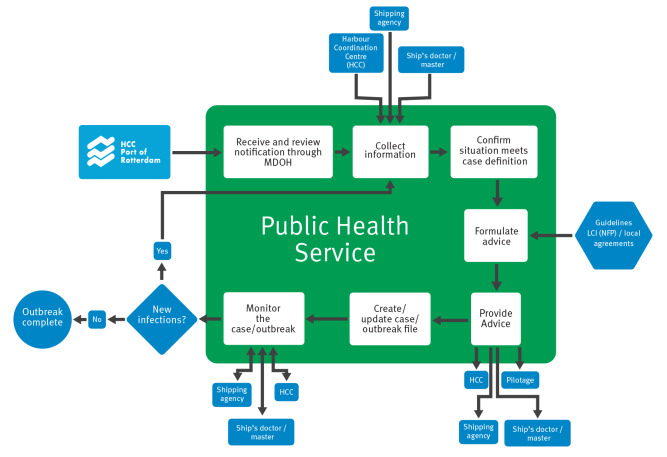
Process for managing a COVID-19 notification by the Public Health Service for vessels arriving at the Port of Rotterdam, the Netherlands, 1 January 2020–31 July 2021

HCC: harbour coordination centre; LCI: Landelijke Coördinatie Infectieziektebestrijding (National Coordination of Infectious Disease Control); MDOH: Maritime Declaration of Health; NFP: National Focal Point.

Guidance to manage COVID-19 on board ship was developed in consultation with stakeholders including PRA, and the PHS at Rotterdam and nationally [19]. It was first published on 14 April 2020 with more detailed guidance separately for cruise ships [20]). The guidance described measures to be taken on board in case of suspected COVID-19 infection, regarding case management, source and contact tracing, testing, isolation, quarantine and communication. The workflow for partners of PRA is shown in Figure 1. Once a suspected case was notified to the PHS, details were entered manually into HPZone, an electronic file where details on suspected cases, related source and contact tracing information are documented. Contact was made with the ship’s agent, captain or ship’s physician and the situation on board was inventorised, i.e. the condition of the patient(s) (symptoms, comorbidities, date of onset and duration of illness), their close contacts (location of boarding / disembarkation, interpersonal contacts among crew on board), rank and function of case and contacts, sanitation measures taken on board and information about the ship (current location, previous or planned route). Notably, medical history on individual crew members and other clinical data beyond that required by PH guidelines were not systematically recorded in HPZone.

On receipt of a notification of suspected COVID-19, the circumstances on board the vessel were reviewed and information collected by the Public Health Service as available from the harbour coordination centre or the ship / ship’s agent. If COVID-19 was confirmed on board, advice on appropriate outbreak control measures was formulated by the Public Health Service, per protocol, and provided to relevant stakeholders as illustrated.

### Data management

COVID-19 case data extracted from HPZone included date of illness onset, date of notification on board, total crew count, number of cases confirmed after the outbreak investigation process, related hospitalisations and deaths.

To derive a notification rate of COVID-19 among vessels arriving at the port, arrival data were extracted from the port’s proprietary administrative system, the Harbour Management and Information System (HaMIS). A 13 digit alpha-numeric unique call reference number (UCRN) was used to derive a denominator of new arrivals, and the international maritime organisation (IMO) number, a number unique to each vessel, was used to identify individual ships [21]. Other variables extracted from HaMIS included vessel type, year of build, last port visited and date of arrival at port. Vessels were categorised by function or type of consignment as follows: (i) workships, which included work or repair vessels, offshore support vessels, tugs and dredgers; (ii) tankers, carrying liquid in bulk, liquid nitrogen gas (LNG) and oil; (iiI) cargo ships, including general cargo, container and bulk carrier vessels; (iv) Passenger ships, including cruise ships, ferries and yachts; and (v) other, including fishing and navy vessels. The year of build of the ship was recorded, as the age of the vessel could be considered a proxy measure, reflecting the ability to implement control measures or sophistication of ventilation systems, for example. Last port and country of call was recorded using the UN/LOCODE, a 5-character Code for Trade and Transport Locations. Harbour Management and Information System data were linked manually to HPZone data using the IMO number, cross referenced with the name of the ship and date of COVID-19 case notification to link to the correct arrival at port (denoted by the UCRN).

### Statistical analysis

Vessel characteristics were described using frequencies and percentages. The COVID-19 attack rate (AR) on board was defined as the proportion of confirmed cases among the crew. The notification rate of COVID-19 on board vessels with an assigned UCRN was the number of arrivals at port where a COVID-19 event was notified, divided by the total number of unique arrivals at port. Differences in proportions were tested using Person’s chi-squared test and in medians using the Wilcoxon rank-sum test (2 independent groups) or the median test (> 2 independent groups). Correlation of quantitative variables was examined using a scatterplot and Pearson’s correlation coefficient, considered significant at p<0.05.

### Selection of case studies

Data collected during routine surveillance did not allow an in-depth analysis of the factors contributing to on-board transmission, although a common understanding among those involved was that on-board conditions may favour this [22,23]. In practice, PHS guidance was to tailor advice to each vessel and event [19]. Two case studies were arbitrarily selected to illustrate the outbreak management process and the complexity of COVID-19 control posed by the ships’ environment (Box 1 and 2). Qualitative information on outbreak control was extracted from HPZone.

Box 1 **Ship 1**

A workship with an on-board crew of 158, docked in the Port of Rotterdam in March 2021 for annual maintenance. On 11 April, one symptomatic crew member tested positive for COVID-19. On 12 April, all crew members were screened by a commercial laboratory and 44 asymptomatic crew were confirmed positive. Most of these crew members (29/44) developed symptoms 3 days later, on 15 April. It was not feasible to isolate all positive cases on board so all cases were disembarked for isolation in a hotel onshore. Crew members testing negative were quarantined on board, leaving their 1–3 person cabins only for essential duties. Subsequently, to allow for deep cleaning of the ship, all crew members, except an essential skeleton crew, disembarked and were quarantined in a hotel onshore during the weekend of 17–18 April. Crew members were quarantined for 5 days and returned to the ship pending a negative test. Overall, 69 of 158 crew members tested positive (AR 44%) and were accommodated onshore in a designated hotel. Four cases were admitted to a hospital, one of whom was admitted to the intensive care unit (ICU). There were no deaths. Communication between the ship and the health department initially ran indirectly through a shipping agency. After 4 days in which insufficient information was received to obtain a full overview of the outbreak and the ship’s outbreak management plan, a digital conference including representatives of the Rotterdam Harbour Coordination Centre, Dutch immigration and the Public Health Service was held in direct consultation with ship’s management. Direct communication with the ship subsequently facilitated regular situation updates and mutual information exchange. Under Dutch law, health and governmental authorities cannot appoint quarantine or isolation locations, and expectations over the quality of accommodation provided differed between the shipping company and Dutch authorities. Ultimately, the shipping company accommodated their crew onshore. A resulting recommendation is to agree a framework for provision of alternative accommodation for quarantine and isolation that can be shared in advance with shipping companies and accommodating parties (e.g. hotels or other vessels).

Box 2 **Ship 2**

Ship 2 was a heavy lift vessel that had an on-board crew of 259 people when it docked for maintenance and repairs at the Port of Rotterdam in December 2020. On 2 March 2021, three crew members had symptoms consistent with COVID and were confirmed PCR-positive. The suspected source was a contractor who tested antigen-negative before boarding the ship in late February 2021, but who subsequently tested positive. Contact tracing on board suggested widespread possible exposure among the crew. In collaboration with the ship’s management, outbreak control measures were implemented, including isolation of those with a positive test result, sanitation measures (disinfection and decontamination), restricted crew movement and systematic testing of the entire crew on alternate days. Test results were communicated to the PHS of the larger Rotterdam Region. Quarantine was advised for direct contacts of infected crew members, but case numbers continued to increase. Limiting factors identified included: (i) before the outbreak some of the 2–4 person cabins housed two crew members working on opposite shifts. When the outbreak started, all crew were given a cabin for their sole use; (ii) the ship’s air recirculation system, installed during ship’s construction in the mid-1980s, could promote transmission. Despite intensive review and excellent working relations between the ship’s management and the health authorities, over 100 crew members tested positive within 2 weeks. Infected crew members were placed in isolation in a hotel in the area, while close contacts remained on board. Nine individuals were admitted to hospital (three to ICU), and 31 were temporarily admitted to a nursing home for additional care. Most crew members made a full recovery (n = 105), while one crew member with an underlying health condition died due to COVID-19. The ventilation system was ultimately replaced and extensive adjustments were made during daily operations. No further outbreaks have since occurred on board this ship or other ships by the same owner / management.

## Results

### Description of port arrivals

**Figure 2:**
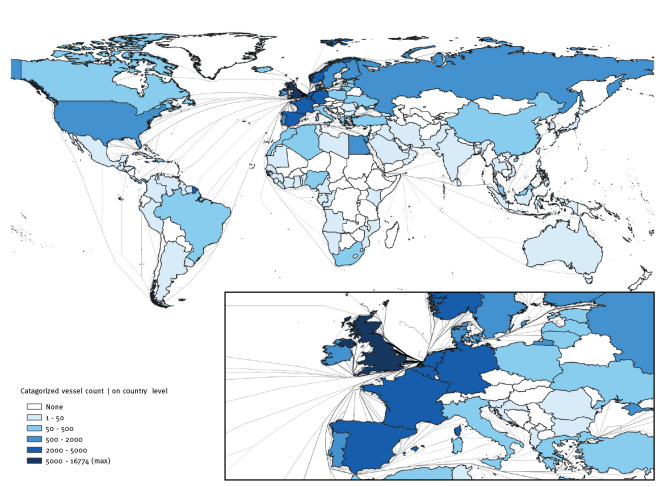
Last port of call for ships arriving at the Port of Rotterdam, the Netherlands, 1 January 2020–31 July 2021 (n = 45,030)

During the study period there were 45,030 registered new arrivals at the Port of Rotterdam (Table 1) by 7,565 individual vessels. Sixty percent of arrivals (n = 26,992) were cargo consignments, 30% were tankers (n = 13,439), 5% (n = 2,360) passenger vessels, and 5% (n = 2,178) workships. The remainder (n = 61) were ‘other vessels’ (e.g. fishing or navy arrivals). The weekly median number of arrivals was 549 (range 448–598), and there were no consistent time trends in the number of weekly arrivals overall or per type of arrival (Supplementary Figure S1). The previous ports before arrival in Rotterdam were distributed across 119 countries, relating to 1,243 ports globally (Figure 2).

**Table 1:** Summary of characteristics of vessels arriving at the Port of Rotterdam, the Netherlands, 1 January 2020– 31 July 2021 (n = 45,030)

Characteristics of vessel arrivals at port	Cargo		Tanker		Passenger		Workship		Other		Total	
Number of arrivals per week, median (range)	329	(104–362)	164	(60–201)	29	(8–33)	26	(6–42)	2	(1–4)	549	(448–598)
Origin/ previous port	n	%	n	%	n	%	n	%	n	%	n	%
Europe	24,607	91.2	11,813	87.9	2,353	99.7	2,102	96.5	61	100	40,936	90.9
Africa	523	1.9	364	2.7	0	0	9	0.4	0	0	896	2
Asia	631	2.3	139	1	0	0	21	1	0	0	791	1.8
North America	713	2.6	635	4.7	7	0.3	27	1.2	0	0	1,382	3.1
South America	195	0.7	153	1.1	0	0	10	0.5	0	0	358	0.8
Middle East	281	1	333	2.5	0	0	9	0.4	0	0	623	1.4
Oceania	42	0.2	2	0	0	0	0	0	0	0	44	0.1
**Total**	**26,992**	**100**	**13,439**	**100**	**2,360**	**100**	**2,178**	**100**	**61**	**100**	**45,030**	**100**

Overall, 91% of arrivals (n = 40,936) were from Europe: 39% (n = 16,129) of arrivals were from the UK (predominantly Harwich, Felixstowe, Immingham), 9% (n = 3,781) from Belgium (mainly Antwerp), 9% (n = 3,555) from other Dutch ports, 8% (n = 3,258) from Germany and 6% (n = 2,367) from Norway.

Global distribution of arrivals by vessel type is in Table 1. Per vessel, the median number of arrivals at the port during the study period was 2 (range 1– 560). The median year of build for vessels overall was 2008 (interquartile range (IQR): 2002– 2012), ranging from the oldest vessels in the category ‘other’ (navy and fishing vessels) with a median year of build of 1993 (IQR: 1987– 2003) to workships, which were constructed most recently (median 2010, IQR: 2004–2014).

### COVID-19 on board ship

**Figure 3:**
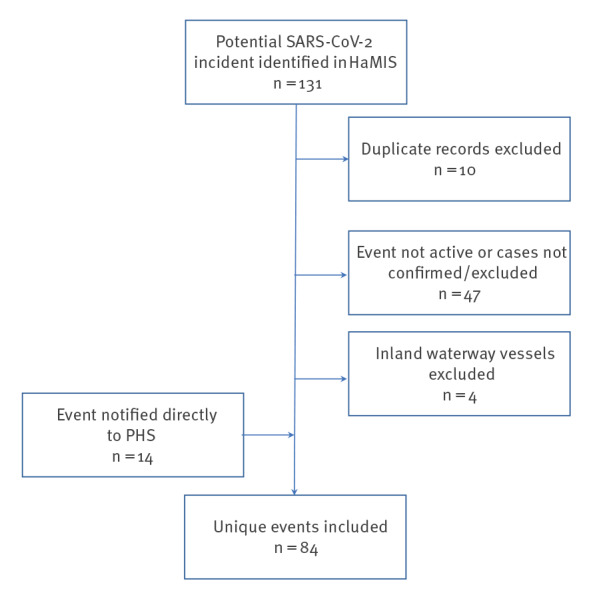
Inclusion and exclusion criteria of COVID-19 cases on vessels docked at the Port of Rotterdam, the Netherlands, 1 January 2020–31 July 2021

HaMIS: Harbour Management and Information System; PHS: Public Health Service.

Overall, a COVID-19 event on board was notified 84 times (Figure 3), involving 622 cases.

**Figure 4:**
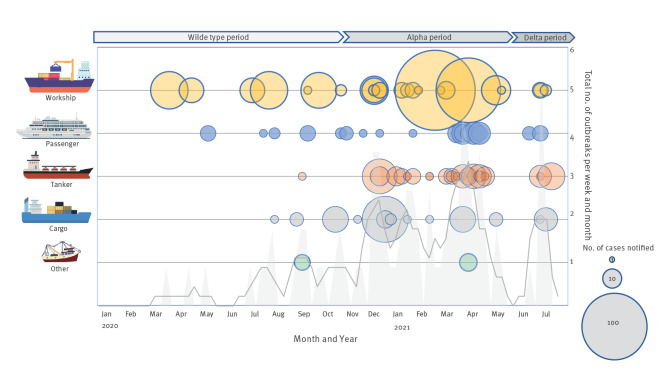
Total number of reported COVID-19 cases notified on board each ship at the Port of Rotterdam by month, year, vessel type and dominant circulating SARS-CoV-2 variant, the Netherlands, 1 January 2020– 31 July 2021

The first notification was received on 31 March 2020 onboard a workship returning to the Port of Rotterdam [9]. The number of events notified per week peaked in the second quarter (Q2) of 2021, subsequently decreasing before a further increase in July 2021 (Figure 4). Four vessels notified two separate events on board.

SARS-CoV-2: severe acute respiratory syndrome coronavirus 2.

The shaded grey area represents the total number of notifications of COVID-19 events experienced on board ship, received per week during each calendar month. The solid grey line is the 4-week moving average summarising the weekly data. The size of the circle indicates how many cases were confirmed per arrival reporting any case of COVID-19.

Overall, 26% (n = 22) of events concerned a single confirmed case on board. After completing the outbreak management process (Figure 1), the median number of cases per event was 4 (IQR: 1–8, maximum of 106 cases). The median AR among the crew was 17% (IQR: 6.3 44.9). Of 622 cases identified, 2.7% were hospitalised (n = 17) and 0.5% died (n = 3) (Table 2).

**Table 2:** COVID-19 events occurring on board ships docked at the Port of Rotterdam by vessel type, with associated attack rates, hospitalisations and deaths, the Netherlands, 1 January 2020–1 July 2021

Vessel type	Number of arrivals reporting COVID-19 on board		COVID-19 cases on board ship				AR		Hospitalisations		Deaths	
			Total crew on board		Total cases		Median	IQR				
	n	%	n	%	n	%			n	% of total cases	n	% of total cases
Cargo	18	21.4	303	6.2	69	11.1	24.8	10.5–42.1	0	0.0	1	1.4
Tanker	25	29.8	450	9.1	121	19.5	28.6	8.7–45.4	4	3.3	0	0.0
Passenger^a^	13	15.5	2,151	43.7	84	13.5	2.5	0.8–11.2	2	2.4	0	0.0
Workship	26	31.0	2,010	40.8	339	54.5	26.4	6.7–43.7	11	3.2	2	0.6
Other	2	2.4	10	0.2	9	1.4	90	80.0–100.0	0	0.0	0	0.0
Total	84	100.0	4,924	100.0	622	100.0	19.1	6.9–43.9	17	2.7	3	0.5
Pandemic period												
Wildtype	16	19.0	1,090	22.1	121	19.5	14.5	6.5–30.9	2	1.7	1	0.8
Alpha	58	69.0	2,557	51.9	454	73.0	16.7	5.0–44.4	14	3.1	2	0.4
Delta	10	11.9	1,277	25.9	47	7.6	34.8	9.1–50.0	1	2.1	0	0.0
Total	84	100.0	4,924	100.0	622	100.0	19.1	6.9–43.9	17	2.7	3	0.5

AR: attack rate; IQR: interquartile range.

^a^ On board vessels in the passenger ship category, cases among crew only were notified during the study period.

COVID-19 statistics by vessel type are shown in Table 2. Crew on board passenger ships and workships accounted for 44% and 41%, respectively, of the total crew on COVID-19 stricken vessels. The AR range on all vessel categories was wide (from 0.8% to 100%). The median AR on cargo vessels, tankers and workships ranged from 25% to 29%. In absolute terms, 55% of cases (339/622) occurred on workships. By contrast, the median AR among crew on passenger ships was 2.5%, ranging from 0.7% to 16.7% on board cruise ships (n = 6), 0.8 to 47% on ferries (n = 5) and 2.9% to 46% on yachts. Notably, no passenger cases were notified during the study period when passenger travel was restricted. The difference between the median by vessel type and the overall median was not statistically significant (p = 0.068). During calendar periods when wildtype and subsequently Alpha variants dominated, the median AR was similar, reaching a maximum during the Delta dominant period. Differences were not statistically significant (median test, p = 0.765, Table 2).

Where previous port before arrival in Rotterdam was reported by ships experiencing a COVID-19 event (new arrivals only, 78/84), 83% originated in Europe, mainly from the UK (29%, n = 19), other ports in the Netherlands (n = 17, 26%), Norway (n = 9, 14%), France (n = 3, 5%), Portugal (n = 3, 5%) and Belgium (n = 2, 3%). Vessels coming from North America accounted for 8% (n = 6) and other global regions, each less than 3%. Of 84 vessels with COVID-19 on board, 16 were exempt from filing the date of previous port visit (e.g. 9 ferry roll-on-roll-off arrivals that make repeated daily crossings on the same route, and seven small fishing vessels that do not leave the port for longer than 24 hours). Dates were not reported by the shipping agent in four additional cases. Of the remaining 64 arrivals, 28% of COVID-19 events were notified before or on the day of arrival, 27% within 1–4 days post arrival, 6% within 5–14 days and the remainder at least 15 days after arrival at port (Supplementary Figure S2).

The distribution of time-to-notification of COVID-19 events differed by vessel type (p = 0.007), with 51% of early notifications (before and/or within 1–4 days of arrival at port) from tankers. Of late COVID-19 notifications (> 30 days post arrival – usually vessels docked for maintenance), 75% were workships. Among vessels notifying an event within 1–14 days post arrival that were potentially at sea during the transmission period (n = 21), correlation between the number of days at sea before arrival in Rotterdam and the AR on board, although statistically significant, was not strong and data points were widely dispersed (p = 0.033, Supplementary Figure S3). There was no relationship between the AR and the number of previous ports visited in the 28 days before arrival.

### Notification rate of COVID-19 among new arrivals at the port

Of 84 COVID-19 events notified, 78 were new arrivals assigned a UCRN registration number during the study period, and for which a denominator could then be derived. This yielded a notification rate of 173 per 100,000 arrivals (78/45,030*100,000), occurring on 1% of vessels (78/7,565). Of the remaining six events, four were UCRN exempt (< 500 gross tonnage) and one vessel that had two outbreaks on board was already in port on 1 January 2020.

Comparing arrivals with and without COVID-19 cases on board, the notification rate differed by the type of vessel arrival, being highest among workships (1.01%) and lowest in cargo vessels (0.07%, p = 0.0000). The median year-of-build (YOB) did not differ overall, except among passenger ships where those reporting a COVID-19 event were older (median YOB 2001, IQR: 1989–2007, n = 13) compared with those not reporting COVID-19 (median YOB 2010, IQR: 2001–2011, n = 2,345, p = 0.0029 (Supplementary Figure S4). Distribution of last port-of-call before arrival in Rotterdam did not differ.

### Outbreaks on board: two case reports from the Port of Rotterdam

Two case studies illustrating the complexity of outbreak control on board are presented in Box 1 and 2. Initially, advice provided to ships included that a case should isolate in a single cabin and close contacts were advised to quarantine on board. Only essential personnel were allowed to (dis)embark, and newly embarking crew were tested. In practice, this was not always possible as illustrated in the first case study. The outbreak evolved rapidly and the accommodation capacity on board was insufficient to allow for single cabin isolation and quarantine. Ultimately, 44% of the crew tested positive and both cases and contacts were accommodated onshore. For crews of non-EU nationality, this had to be done in consultation with the Department of Immigration. Sourcing suitable accommodation onshore was the responsibility of the shipping agent.

The second case study (Box 2) highlights how, even with an optimal working relationship between the shipping agent and public health authorities, transmission still occurred resulting in large outbreak with serious morbidity and one death. Specific guidance was developed for safe transport of a positive case to shore [24]. Over time, a specialised outbreak control team was trained to advise ships / shipping agents regarding testing, isolation and quarantine, and communicate this advice with relevant partners within the Port Health Authority of Rotterdam (PHAR).

## Discussion

We assessed the frequency and magnitude of COVID-19 on ships at the Port of Rotterdam, the largest port in Europe. During the study period, ships continued to arrive, albeit with smaller shipments in the first half of 2020. Even as the number of arrivals was sustained, a low notification rate of COVID-19 events on board arrivals at port was recorded (173/100,000 arrivals, on 1% of vessels, with higher AR and caseloads on workships than on other vessel types). Temporal trends overall mirrored global epidemiology: more outbreaks and elevated AR were observed as the wildtype virus gave way to variants of higher transmissibility (Alpha and Delta).

The low notification rate observed overall is plausible, although likely underestimated. Guidance on outbreak prevention was issued by the International Chamber of Shipping in March 2020 [25]. Additionally, many countries implemented port entry restrictions [26], requiring a negative PCR or antigen test before boarding and subsequently, vaccination. Travel restrictions and border closures meant that crew repeatedly extended their contracts (typically lasting 3–9 months), thus reducing crew turnover [27] and the potential for viral introduction on board. Passenger vessels, particularly cruise liners, faced more stringent restrictions [20,28] and stopped transporting passengers from March 2020 [29] until August 2020. Dutch ports were also intermittently closed to cruise ships (from December 2020 to July 2021 and December 2021 to January 2022). Ferries continued to sail, but without passengers on board, and no cases among passengers were notified during the study period. Tracking of passengers on ferries by the PHS is not conducted as passengers (dis)embark at regular, short intervals (often several times daily) and cases may indeed have occurred as normal operations resumed.

Among the crew, under-ascertainment of cases was undoubtedly a problem, as suggested by the relatively high proportion of cases hospitalised (2.7%) and deaths (n = 3). Few events were notified overall until Q3 of 2020, except among workships. Workships generally have large crews (high contact rates and potential for transmission), high crew turnover due to changing specialist skill requirements (opportunities for viral introduction) and often have access to medical expertise and / or testing on board not available on other vessels. Conversely, the AR among crew on passenger ships was lower than on other vessel types, possibly reflecting better infection control experience and capacity, and was broadly consistent with the ARs reported elsewhere on cruise ships [30]. Anecdotally, a more conservative approach to testing was taken on ships experiencing an outbreak, sometimes retesting contacts daily rather than at 5-day intervals as recommended by the PHS (Personal communication: E. Gebuis, 20 April 2022). This information was not routinely documented, however, and we cannot say whether there were systematic differences in testing by vessel type. Shipping companies typically used commercial testing laboratories that are obliged to notify positive results to the PHS [15], but there is no legal basis for shipping agencies themselves to report test results, positive or negative, to the PHS. Sharing of samples for sequencing, a key component to understand viral introduction and transmission [31], also varied depending on the commercial facility. In the absence of accessible samples, enhanced surveillance of sewage sampling at port could be an alternative means to give an early indication of viral activity at port and provide information for preventive action [32]. Regarding the confirmed cases, we did not have access to individual-level demographic or clinical data, and thus could not stratify patient risk. Absolute case numbers were low and the number of hospitalisations and deaths reported to the PHS may also be incomplete.

The case studies were chosen to illustrate the rapidity at which outbreaks on board can escalate, particularly for a respiratory disease of short generation time [13]. As illustrated, adequate space for isolation and quarantine on board and the potential to disembark are key capacities [33,34]. Older passenger vessels experiencing an outbreak had higher ARs than newer ones, perhaps reflecting more ergonomic interiors and ventilation systems. As in the case of repeat outbreaks, environmental sampling and detailed strain information would have helped to shed light on whether environmental contamination was implicated in (re)infections [35], but also on the role of new viral introductions or super spreader events.

The study was enabled by the willingness of PHAR partners to share data and information and reflects the first use of PRA administrative data to quantify outbreak activity on board ship. There were several limiting factors, however. Administrative datasets are not designed for research purposes and relevant data were distributed across numerous databases and stakeholders. All data extraction from the PHS database (HPZone) was done manually, which was time consuming and potentially prone to omission or error. There was no single identifier across both systems and data linkage was also conducted manually. Established identifiers such as the UCRN and IMO number allowed us to reliably identify both vessels and arrivals, but vessels commuting Dutch inland waterways, registered under a different system, were excluded. Although some vessels were exempt from filing an MDoH or did not require registration on arrival, this number was negligible to the overall result (Personal communication: R. de Vries, 05 April 2022).

Advance agreement on data sharing protocols and processes between port authorities, PHS and clinics / hospitals and digitisation of data collection systems (HPZone in particular - the MDoH has been submitted digitally since 1 September 2021 [36]), would help to ensure a minimum set of common data elements within databases and facilitate efficient data linkage and more agile and informative surveillance and response locally. Additionally, in advance of outbreaks, engagement between public health and port authorities, shipping agencies and others such as hoteliers and immigration if onshore control measures are required, would also facilitate planning for disembarkation, should it be necessary. At the European level, national authorities adhere to different port entry restrictions and regulations. Extension of the EU Healthy Gateways Joint Action project (www.healthygateways.eu) would help to further evolve a common framework in which to formulate port entry policies, and a more integrated use of the EU web-based networks and tools (e.g. EU Common Ship Sanitation Database [37]) for rapid information exchange.

## Conclusion

COVID-19 events were notified infrequently on board ships at the Port of Rotterdam but under ascertainment is likely. Since September 2021, all seafarers, irrespective of their nationality, are offered COVID-19 vaccination at the port and ca 30,000 vaccines have been administered. Collaboration between different disciplines nationally and at EU level, as proposed, will help to ensure our resilience against future health threats that impact maritime transport, as they continue to emerge.

## Supplementary Data

225000 Supplementary Material
