# Supplementary material for: COVID-19 outbreaks among crew on commercial ships at the Port of Rotterdam, the Netherlands, 2020 to 2021
Source: Euro Surveill. 2023 Apr 20;28(16):2200525. doi: 10.2807/1560-7917.ES.2023.28.16.2200525 (PMC10283453; doi:10.2807/1560-7917.ES.2023.28.16.2200525)
Supplement: Supplementary Material [file 22-00525_WHELAN_Supplementary_material.pdf]

This supplementary material is hosted by *Eurosurveillance* as supporting information alongside the article 'COVID-19 outbreaks among crew on commercial ships at the Port of Rotterdam, the Netherlands, 2020 to 2021', on behalf of the authors, who remain responsible for the accuracy and appropriateness of the content. The same standards for ethics, copyright, attributions and permissions as for the article apply. Supplements are not edited by *Eurosurveillance* and the journal is not responsible for the maintenance of any links or email addresses provided therein.

Supplement S1. Designation of 'pandemic periods' in which known variants were dominant

'Pandemic periods' were defined according to calendar period when variants of concern were known to be dominant in Europe (from where most shipping traffic arrived). The B.1.1.7 (alpha) variant first emerged in the UK in September 2020 but was not in community circulation until November 2020. The B.1.617.2 (delta) variant emerged first in India in October of 2020, but was also not widely in circulation until mid-2021. On these bases, we designated the period from the start of the study until 01 November 2020 the 'wildtype period', from 01 November 2020 until 01 June 2021, the 'alpha-dominant' period and from 01 June 2021 until 31 July (the end of data collection), the 'delta-dominant' period.

Supplement S2. Sample Maritime Declaration of Health

Maritime Declaration of Health

To be completed and submitted to the competent authorities by the masters of ships arriving from foreign ports.

**Submitted at the port of:**	**Date**:
**Name of ship or inland navigation vessel:**	**Registration/IMO No.**
**Arriving from:**	**Sailing to:**
**(Nationality) (Flag of vessel):**	**Master's name:**
**Gross tonnage (ship):**	**Tonnage (inland navigation vessel):**

Valid Sanitation Control Exemption/Control Certificate carried on board? Yes ☐ No ☐
Issued at: **Date:**

Reinspection required? Yes ☐ No ☐

Has ship/vessel visited an affected area identified by the World Health Organization? Yes ☐ No ☐
Port: **Date of visit:**

List of ports of call from commencement of voyage with dates of departure, or within past 30 days, whichever is shorter:

Upon request of the competent authority at the port of arrival, list crew members, passengers or other persons who have joined ship/vessel since international voyage began or within past 30 days, whichever is shorter, including all ports/countries visited in this period (add additional names to the attached schedule):

(1) **Name:**	joined from: (1)	(2)	(3)
(2) **Name:**	joined from: (1)	(2)	(3)
(3) **Name:**	joined from: (1)	(2)	(3)

Number of crew members on board: 120

Number of passengers on board:111

**HEALTH QUESTIONS**	**Yes**	**No**
1. Has any person died on board during the voyage otherwise than as a result of accident?<br>If yes, state particulars in attached schedule.<br>Total number of deaths:	☐	☐
2. Is there on board or has there been during the international voyage any case of disease which you suspect to be of an infectious nature?<br>If yes, state particulars in attached schedule.	☐	☐
3. Has the total number of ill passengers during the voyage been greater than normal/expected?<br>How many ill persons:	☐	☐
4. Is there any ill person on board now?<br>If yes, state particulars in attached schedule.	☐	☐
5. Was a medical practitioner consulted?<br>If yes, state particulars of medical treatment or advice provided in attached schedule.	☐	☐
6. Are you aware of any condition on board which may lead to infection or spread of disease?<br>If yes, state particulars in attached schedule.	☐	☐
7. Has any sanitary measure (eg, quarantine, isolation, disinfection or decontamination) been applied on board?<br>If yes, specify type, place and date:	☐	☐
8. Have any stowaways been found on board?<br>If yes, where did they join the ship (if known)?	☐	☐
9. Is there a sick animal or pet on board?	☐	☐

Note: In the absence of a surgeon, the Master should regard the following symptoms as grounds for suspecting the existence of a disease of an infectious nature:

(a) fever, persisting for several days or accompanied by (i) prostration; (ii) decreased consciousness; (iii) glandular swelling; (iv) jaundice; (v) cough or shortness of breath; (vi) unusual bleeding; or (vii) paralysis

(b) with or without fever: (i) any acute skin rash or eruption; (ii) severe vomiting (other than sea sickness); (iii) severe diarrhoea; or (iv) recurrent convulsions.

I hereby declare that the particulars and answers to the questions given in this Declaration of Health (including the Schedule) are true and correct to the best of my knowledge and belief.

Signed --- Countersigned --

Master Ship's Surgeon (if carried)

Date:

Supplement S.3 Reporting exemptions at the Port of Rotterdam

The text below is translated from the national Dutch guidance 'COVID-19 on ships in ports'.[1]

The Maritime Declaration of Health (MDoH) must be actively requested by the port authorities for every ship that makes an international voyage and from fishing vessels that make a multi-day voyage. Ships not on an international voyage (not calling at port abroad), with a maximum of 10 persons on board and where there is no crew change at sea or at anchorage, only need to submit an entry MDoH when requested by the port authority. If there is a COVID-19 infection on board, an MDoH must still be handed over to the port authorities. Regionally, the municipal health department (GGD) should agree with the port authorities about a coordinated response in the event of a positive MDoH.

When an MDoH is not yet available or is incomplete, the following information must be actively requested:

- whether there are crew members with complaints that may be related to COVID-19;
- whether measures have been taken on board, such as isolation of persons with complaints appropriate to COVID-19 or quarantine of close contacts (category 1&2);
- what the vaccination status of the crew members / persons on board is.

If tests have been carried out on board, information regarding the specific tests used must be requested:

- what kind of tests (antigen test, or possibly point of care PCR tests);
- when administered and by whom were the tests performed and assessed;

If the MDoH is negative/there are no crew members on board with COVID-19, a licence to enter a port may be granted ('*free pratique*')

- Agree with the port authorities that every mooring ship receives general advice for the prevention of an outbreak of COVID-19 and what to do if there is a suspicion of COVID-19.

[1] COVID on ships and in ports. COVID-19 op schepen en in havens | LCI richtlijnen (rivm.nl)

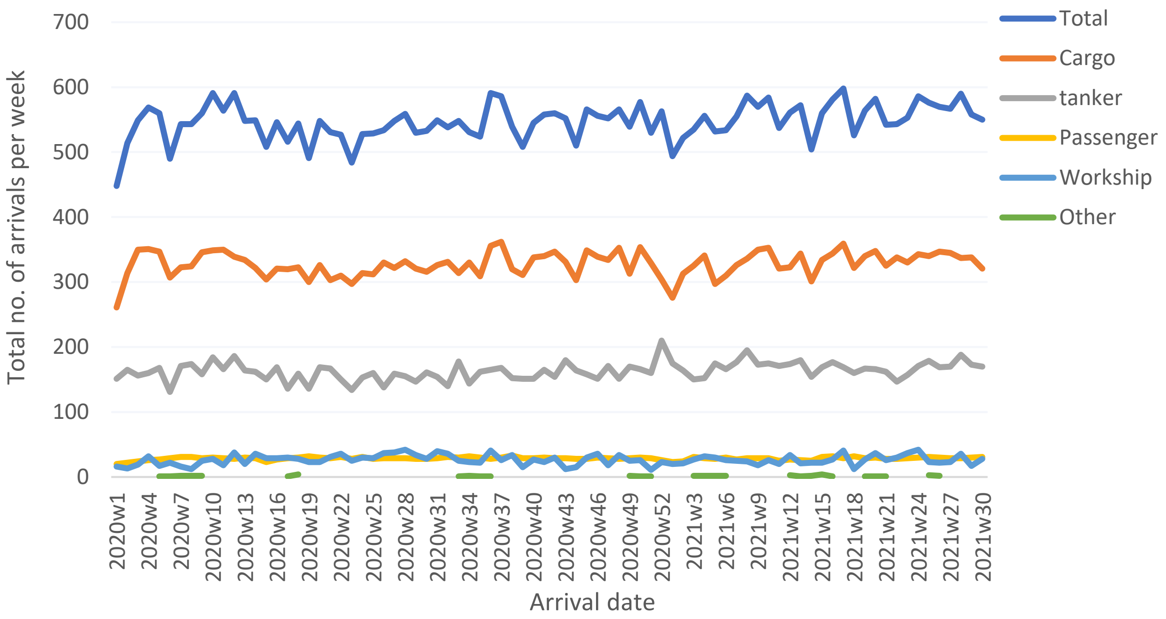

Supplementary Figure S1. Number of weekly arrivals by vessel type, 01 January 2020 until 31 July 2021.
There were no consistent trends in the number of weekly arrivals by vessel type.

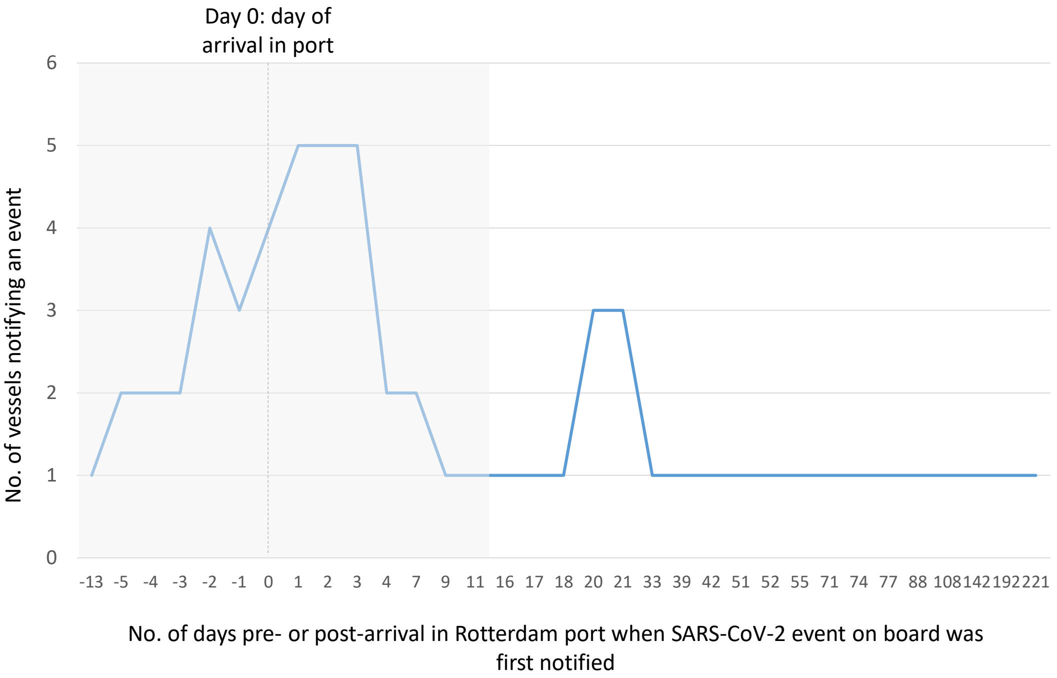

Supplementary Figure S2. Number of days pre or post arrival at port by the number of vessels notifying.

The shaded area highlights the vessels that notified a SARS-CoV-2 event within 14 days of arriving at port. Of 62 events with complete data, 18 notifications were received prior to or on the day of arrival at Rotterdam port. A further 17 were notified within 1 to 4 days of arrival, 4 within 5 to 14 days of arrival, 9 within 15-29 days and 14 after 30 days in port or longer. Vessels that are remain in port for prolonged periods are usually under repair or in receipt of routine maintenance.

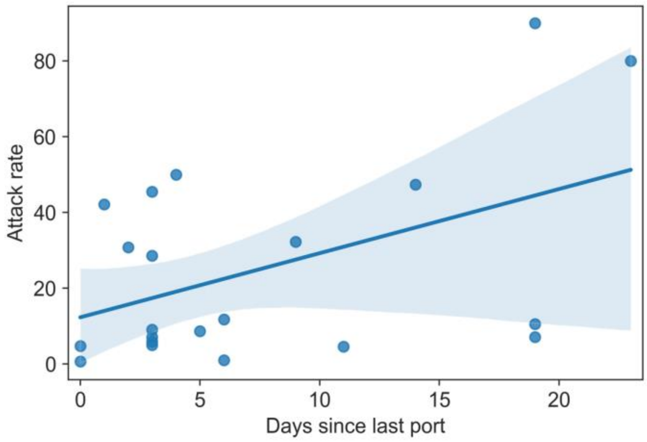

Supplementary Figure S3. Relationship between days at sea since last port of call and prior to arrival at Rotterdam, and the attack rate on board ship among vessels that reported an event from 1 to 14 days after arrival at Rotterdam.

Of 21 vessels included in this analysis, there were 12 tankers (57%), 5 cargo ships, 3 workships and 1 passenger ship (Pearson's correlation coefficient=0.4665, p=0.033)

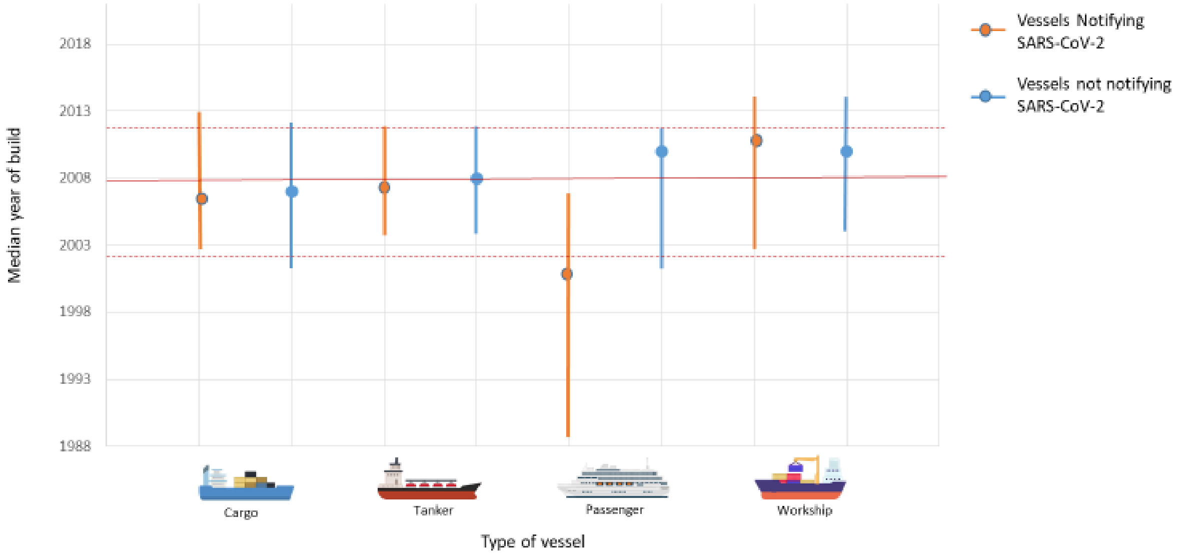

Supplementary Figure S4. Median year of build (and interquartile range) of arrivals at Port of Rotterdam, by vessel category and a SARS-CoV-2 event occurring on board, Jan 2020 to Jul 2021.

There was no overall difference in the median year of build for arrivals notifying SARS-CoV-2 versus those that did not, however, when stratified by vessel type, passenger ships where those reporting SARS-CoV-2 events on board were older (median=2001, IQR 1989 to 2007, n=13) compared to arrivals SARS-CoV-2 was not reported (median 2010, IQR 2001 to 2011, n=2345; Wilcoxon rank-sum z = 2.980, p = 0.0029).
